# Unravel subseafloor hydrothermal leaching and magmatic degassing during chimney formation at Kolumbo volcano

**DOI:** 10.1038/s41598-025-99586-5

**Published:** 2025-04-26

**Authors:** Simon Hector, Qasid Ahmad, Clifford G.C. Patten, Massimo Chiaradia, Paraskevi Nomikou, Stephanos Kilias, Alexandre Peillod, Simon Wagner, Jochen Kolb

**Affiliations:** 1 Institute of Applied Geosciences, Geochemistry and Economic Geology, KIT, Karlsruhe, Germany; 2 Laboratory for Environmental and Raw Materials Analysis, Karlsruhe, Germany; 3 Centre de Recherches Pétrographiques et Géochimiques, Vandoeuvre-lès-Nancy, France; 4 Institute of Mineralogy and Petrography, University of Innsbruck, Innsbruck, Austria; 5 Section of Earth and Environmental Sciences, University of Geneva, Geneva, Switzerland; 6 Department of Geology and Geoenvironment, National and Kapodistrian University of Athens, Athens, Greece

**Keywords:** Geochemistry, Petrology

## Abstract

Hydrothermal chimneys are the upmost expression of fluids, metals and ligands transfer from the subseafloor to the hydrosphere, eventually forming seafloor massive sulfides. In volcanic arc settings, both magmatic and hydrothermal fluids occur together. While each fluid reflects different metal mobilizing mechanisms (i.e. magmatic degassing and hydrothermal leaching of subseafloor lithologies), it is unclear which metals they respectively provide to the budget of the chimneys. We investigate the metal sources and mobilizing mechanisms associated with a gold-rich hydrothermal field from Kolumbo volcano (South Aegean Volcanic Arc, Greece) by comparing Pb isotope ratios of ore minerals from a chimney with those of potential source rocks. Four key findings result from our study: (1) Kolumbo volcanic rocks are the main source of Pb for the chimneys; (2) Magmatic assimilation of Cycladic Basement allows to track magmatic differentiation and identify metal mobilizing mechanisms for Pb and metals with similar behavior. At Kolumbo, magmatic degassing mobilizes As, Ag, Au, Cu, Hg, Sb, Sn and Zn along with Pb, while hydrothermal leaching of rhyolite provides Tl and likely some base metals to the chimneys; (3) Magmatic fluids contributed to galena and Sb-Pb sulfosalts formation while pyrite formed from hydrothermal fluids leaching rhyolite; (4) Galena growth zones in pyrite reveal episodic pulses of magmatic fluids during the chimney growth. The combined use of Pb isotopes on ore minerals and source rocks provides an additional tool to discriminate between magmatic and hydrothermal fluids contribution during seafloor massive sulfide formation, especially in arc settings where magmatic assimilation of crustal material with distinct isotopic signature is more likely to occur.

**Supplementary Information:**

The online version contains supplementary material available at 10.1038/s41598-025-99586-5.

## Introduction

Hydrothermal chimneys, such as “black smokers”, result from large scale hydrothermal cell below the seafloor, where fluids, metals and ligands are transferred from the sub-seafloor to the hydrosphere, leading to seafloor massive sulfide (SMS) formation, dissemination of heavy metals in the environment and development of thriving ecosystems^1^. While in mid-oceanic ridges setting hydrothermal leaching of oceanic crust is the dominant metal mobilizing mechanism^2,3^, magmatic degassing is considered to be a major source of metals during SMS formation in arc environment^4–9^. Deciphering between magmatic degassing and hydrothermal leaching during arc-SMS formation, however, is notoriously challenging as both mechanisms occur together within the same hydrothermal cell system^4,10^. There are numerous geochemical evidences of magmatic fluids causing an enrichment in epithermal elements (i.e. Au, Ag, As, Hg, Sb, Te, and Tl) – e.g. stable isotopes^11–14^, trace elements^9,15^, and fluid geochemistry^10,16^. However, the contribution of hydrothermal leaching to the metal budget during arc-SMS formation is widely overlooked^2,7^. In particular, continental margin arc environments have a wide diversity of leachable lithologies (e.g. volcano-sedimentary, metamorphic, intrusive rocks, etc.) that may influence the metal budget, especially concerning base metals. Nevertheless, hydrothermal leaching alone cannot account for the high content of epithermal elements in arc-SMS, because it would require source regions too large for the hydrothermal system^4^. Therefore, identifying metal sources and distinguishing magmatic degassing and hydrothermal leaching as metal mobilizing processes remain key questions to the understanding of arc-SMS metallogeny^8,13^. The submarine Kolumbo volcano, hosted on a thinned continental margin, offers an ideal setting to tackle these questions thanks to the geochemical signature of igneous rocks being distinct from country rocks. Unlike in intra-oceanic arcs or mid-oceanic setting, here metals mobilized by hydrothermal leaching or magmatic degassing will bear different Pb isotope signature. Comparing in-situ Pb isotope ratios of the Kolumbo Au-rich arc-SMS with those of the different potential sources rocks indicates that hydrothermal leaching of rhyolite mobilizes Tl and likely base metals while magmatic fluids provide epithermal elements and base metals to the chimneys. Growth zones in the chimneys with minerals bearing a magmatic fluid Pb isotope signature (galena and Sb-Pb sulfosalts) reveals that the nature of the mineralizing fluid evolved during the SMS formation, with episodic contribution of magmatic fluid to the hydrothermal system.

## Geological setting

**Fig. 1:**
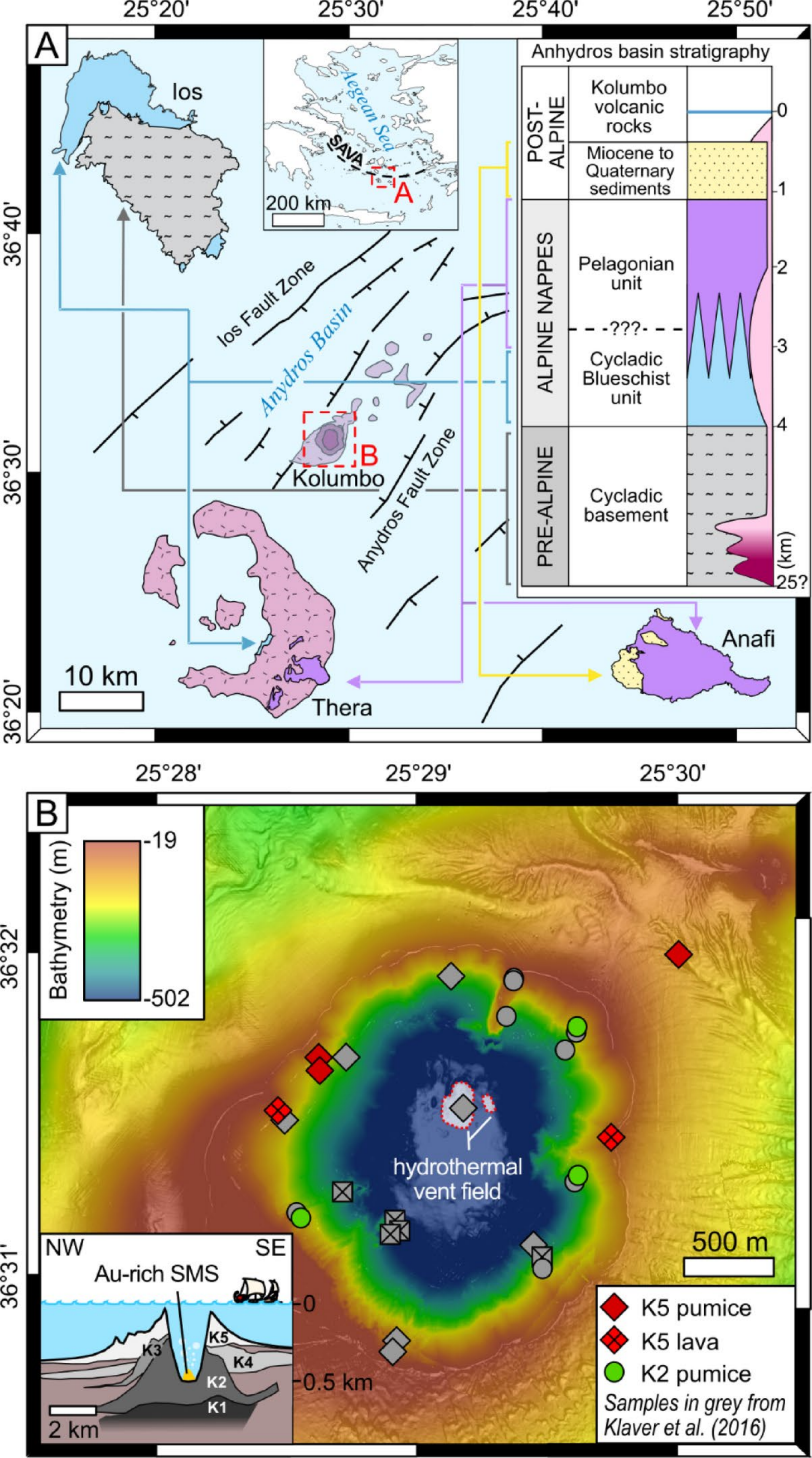
(**A**) Geological setting of the Kolumbo volcano within the Anhydros basin; abbreviation: SAVA = South Aegean Volcanic Arc (modified after refs^20,25–28,31–33^; (**B**) Bathymetric map and stratigraphy of the Kolumbo volcano with samples and Au-rich SMS locations (modified after refs^29,33^.

The submarine Kolumbo volcano belongs to the Christiana-Santorini-Kolumbo volcanic field and is located in the Anhydros rift basin within the South Aegean Volcanic Arc (Fig. 1A)^17,18^. It is located in the center of a half-graben structure where faults (2–3 km) cross-cut the stratigraphy to 2–3 km depth^19,20^. The northern part of the crater hosts a hydrothermal field where boiling CO_2_-rich fluids vent at 265 °C, forming Au-rich polymetallic SMS (Fig. 1B)^21–23^. The source of the mineralizing fluid remain unclear, Cu isotope in pyrite with δ^65^Cu ≈ 0‰ suggest contribution of magmatic fluid^14^, but may also reflect hydrothermal leaching of rhyolite^24^. The stratigraphy below Kolumbo consists of a 10 to 15 km thick, pre-Alpine, continental basement overlain by Alpine nappes and post-Alpine sedimentary rocks (Fig. 1A)^22,25^. The pre-Alpine Cycladic Basement consist of Carboniferous granite, garnet-mica schist and augengneiss, outcropping on Ios^26^. Alpine nappes are divided into two different stratigraphic units: (1) the Cycladic blueschist unit, composed of greenschist to blueschist facies schists and marble, outcropping in the northern part of Ios and at the Athinios harbor in Thera^26,27^; and (2) the Pelagonian unit, composed of greenschist and amphibolite facies rocks, ophiolites with meta-pelagic sequences, marble, flysch and granite, outcropping on the south-eastern part of Thera and on Anafi^27,28^. The presence of the Pelagonian and/or Cycladic Blueschist units below Kolumbo is not certain due to lack of drill-core data^26,27^. Post-Alpine units, Miocene to Quaternary molasse and volcanic rocks, crop out on the western shore of the Anafi island^28^. The volcanic edifice of Kolumbo is composed of a superposition of volcanoclastic units labelled K1 to K5, the later resulting from the 1650 CE eruption (Fig. 1B)^29^. Outcropping units K2 and K5 consist of rhyolitic pumice with minor basaltic to andesitic pumice and lava flows^8,30,31^. The magmatic plumbing system consists of at least two magma chambers (Fig. 1A): (1) a lower crustal one where basaltic-andesitic melt differentiates to rhyolite while assimilating Cycladic Basement^31^; and (2) a rhyolitic upper crustal magma chamber, located between 2 and 4 km depth^32^.

### Samples and analysis

Kolumbo has been sampled in 2010 by the remotely operated vehicle (ROV) Hercules during the cruise NA007 of the exploration vessel^17^. At that time, only samples belonging to the outcropping K2 and K5 units could be collected (Fig. 1B). Both units are geochemically similar and are referred to as Kolumbo volcanic rocks^8^. Samples of the wall rocks below Kolumbo have been collected in outcrops on Anafi, Ios and Thera. Lead isotopes (^204^Pb, ^206^Pb, ^207^Pb, ^208^Pb) of bulk rocks (*n* = 39) and ore minerals (*n* = 30) were analyzed by MC-ICP-MS and in-situ LA-ICP-MS respectively (ESM1).

## Results

### Chimney paragenesis

**Fig. 2:**
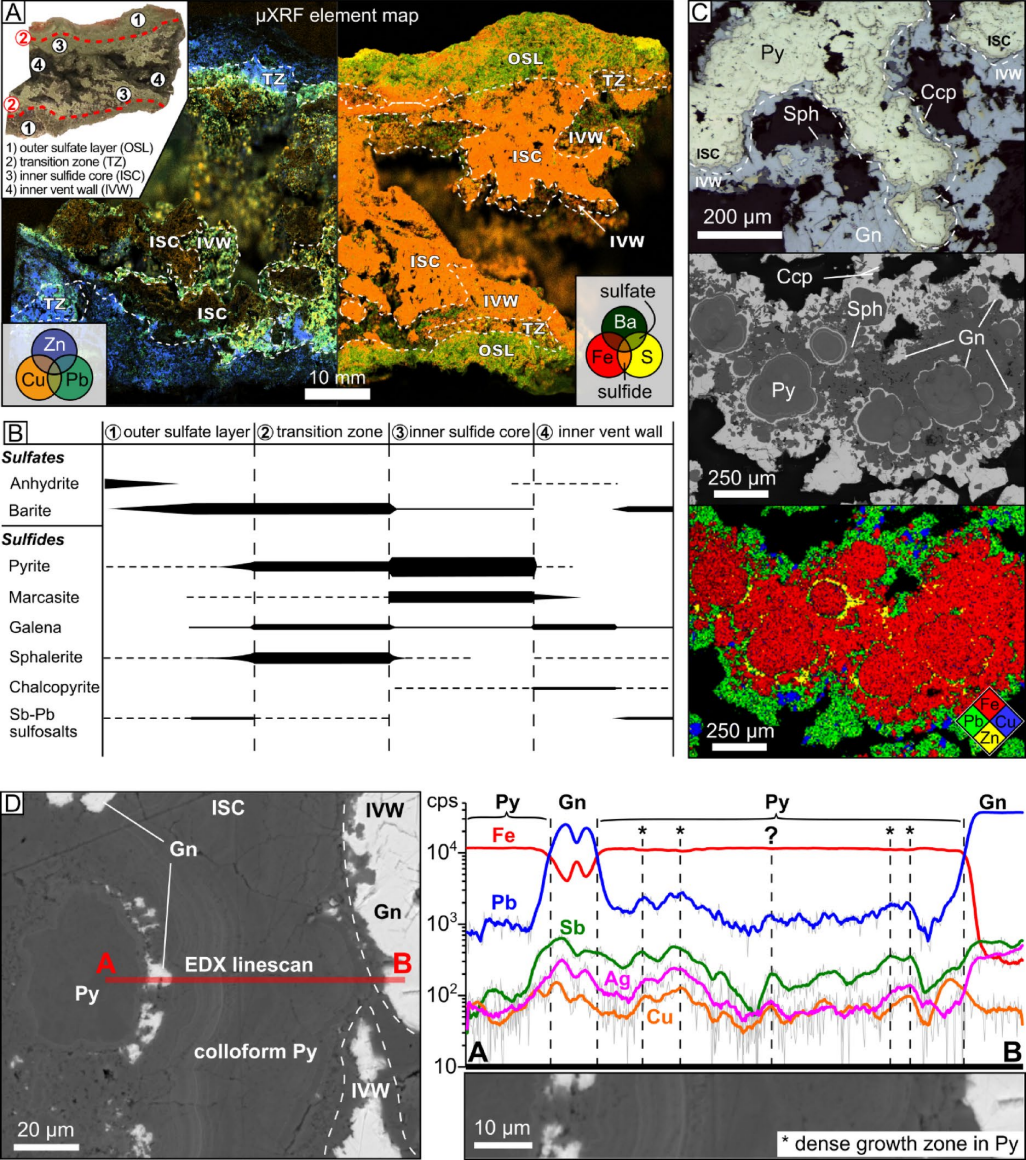
Sulfate-sulfide chimney at Kolumbo. (**A**) Cross cut and µXRF element map of the chimney showing the mineralogical transition from a sulfate-dominated OSL (Ba-rich) to a sulfide-dominated ISC (Fe-rich); (**B**) paragenetic sequence of the sulfate-sulfide chimney; (**C**) Reflected light, BSE-image and XRF element map of the ISC to IVW transition, showing growth zones of galena within pyrite; (**D**) EDX elemental line scan on the ISC to IVW transition from colloform pyrite with galena growth zones (ISC) to galena (IVW). For each element, the signal is smoothed using the adjacent averaging method; the raw signal is displayed in light grey.

The SMS consists of mineralogically zoned sulfide-sulfate chimneys: (1) an outer sulfate layer (OSL) of barite and anhydrite with accessory pyrite, marcasite, galena, sphalerite and Sb-Pb sulfosalts, locally covered by an Fe-rich crust^22^; (2) a transition zone (TZ) of major sphalerite and barite with minor pyrite, galena, marcasite and Sb-Pb sulfosalts; (3) an inner sulfide core (ISC) of major pyrite and marcasite with minor galena, sphalerite, chalcopyrite and barite; and (4) the inner vent wall (IVW) with major galena and barite and minor chalcopyrite, marcasite, pyrite and Sb-Pb sulfosalts (Fig. 2A and B). The OSL, TZ and ISC have a width of 1 to 2 cm, a few mm and 2 to 6 cm, respectively. The zone contacts are transitional, with complex sulfide-sulfate assemblages (Fig. 2A) and textures, such as galena growth zones in pyrite (Fig. 2C). Colloform pyrite displays growth zones of varying composition, with local enrichment in Pb, Sb, Ag and Cu (Fig. 2D).

### Lead isotopes

**Fig. 3:**
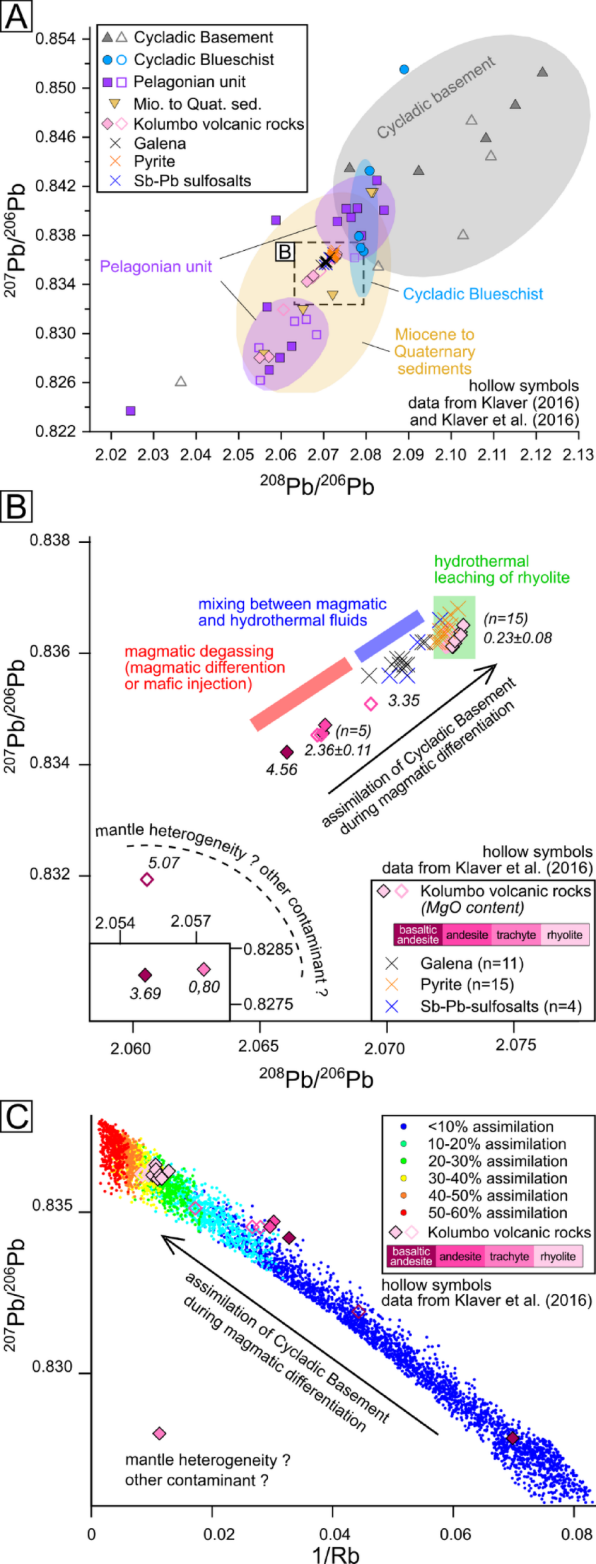
Lead isotope ratios of SMS at Kolumbo and potential Pb source rock units (hollow symbols = data from refs^31,34^, see ESM1). (**A**) ^207^Pb/^206^Pb versus ^208^Pb/^206^Pb plot for galena, Sb-Pb sulfosalts and pyrite and potential source rocks. Pb isotope ratios fields for each lithology calculated using 2D Kernel density graphs containing the data within 1σ; (**B**) Detail of (**A**) showing the shift of Pb isotope ratios of Kolumbo volcanic rocks to higher ^207^Pb/^206^Pb and ^208^Pb/^206^Pb ratios during magmatic differentiation (MgO content from ref^8^; (**C**) Monte Carlo-based modeling of an assimilation–fractional crystallization (AFC) process of Cycladic Basement at Kolumbo using Rb as an incompatible trace element to estimate magmatic differentiation (see ESM2).

Potential source rocks and ore minerals are compared using only ^206^Pb, ^207^Pb and ^208^Pb. We avoid using ^204^Pb as high Hg contents in some pyrites may lead to high isotopic interferences during in-situ Pb isotope analysis that cannot be corrected. The isotope signature of the SMS overlaps with that of Miocene to Quaternary sediments and matches closely that of Kolumbo volcanic rocks (full dataset available in ESM1). Kolumbo volcanic rocks have narrow ^207^Pb/^206^Pb (0.836 ± 0.001) and ^208^Pb/^206^Pb ranges (2.071 ± 0.002) except for three samples with lower ratios (0.828 to 0.832; 2.055 to 2.061) (Fig. 3A-B). Rhyolite has higher ^207^Pb/^206^Pb and ^208^Pb/^206^Pb ratios (~ 0.836; ~2.073) than basaltic-andesite and andesite (~ 0.834; ~2.067) (Fig. 3B). Similarly, the isotope signature of the mineralization appears to be divided into two groups: (1) galena and Sb-Pb sulfosalts (OSL, TZ and IVW, see Fig. 2B) range between andesite and rhyolite; (2) pyrite (ISC, see Fig. 2B) values are similar to rhyolite (Fig. 3B).

## Discussion

### Lead source

The ^207^Pb/^206^Pb versus ^208^Pb/^206^Pb ratio plots show that galena, pyrite and Sb-Pb sulfosalts fall in the broad field of Miocene to Quaternary sediments. However, they also overlap completely with the narrower field of Kolumbo volcanic rocks. While we cannot exclude that the isotopic signature of the ore minerals results from mixing between low and high ^207^Pb/^206^Pb and ^208^Pb/^206^Pb sources (e.g. Pelagonian Unit and Cycladic Basement), the geographic proximity between the SMS and the Kolumbo volcanic rocks strongly points toward the latter as the main source of Pb for the SMS (Fig. 3A).

### Magmatic evolution and crustal assimilation

The Pb isotope ratios of a magma are not affected by magmatic differentiation^35^, thus higher ^207^Pb/^206^Pb and ^208^Pb/^206^Pb ratios in rhyolite than in basaltic andesite suggests crustal assimilation of rocks with higher ^207^Pb/^206^Pb and ^208^Pb/^206^Pb during magmatic differentiation instead of mantle source control (Fig. 3B). The Cycladic Basement is the most likely contaminant as it consistently displays higher ^207^Pb/^206^Pb and ^208^Pb/^206^Pb ratios than the Kolumbo volcanic rocks (Fig. 3A; see also Klaver et al., 2016). Monte Carlo-based modeling of an AFC process indicates that assimilation of up to 20–40% of Cycladic Basement in the lower crustal magma chamber may explain the Rb content and Pb isotope ratios observed in rhyolite (Fig. 3C; ESM2).

### Lead mobilization: magmatic degassing versus hydrothermal leaching

**Fig. 4:**
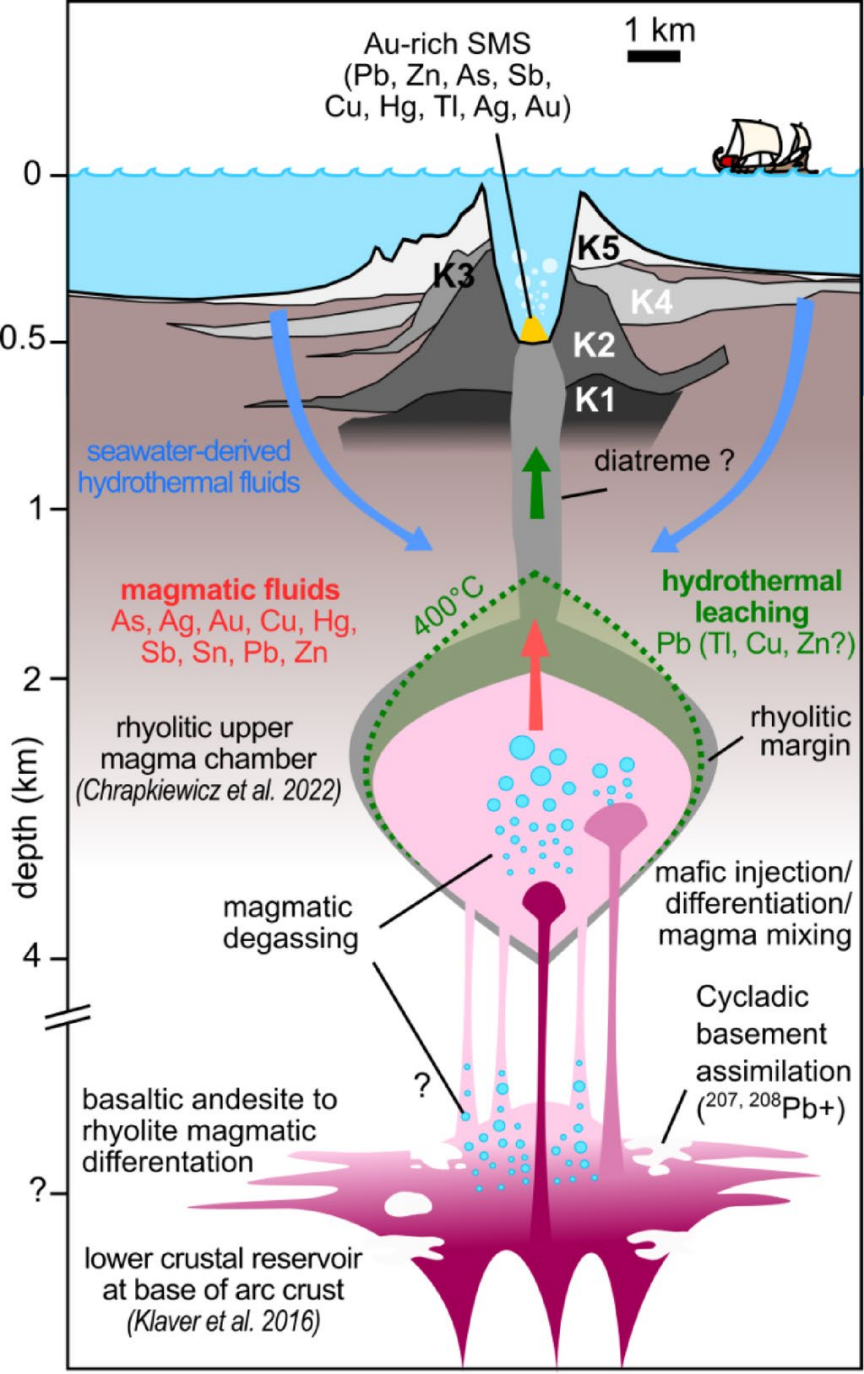
Model of Kolumbo’s magmatic-hydrothermal system and metal mobilizing processes. Magmatic plumbing system based on refs^31,32^, stratigraphy of the K1 to K5 volcanoclastic units based on ref^29^. Magmatic processes and associated metal mobilizing processes based on refs^8,15^.

Volcanic rocks preserve the Pb isotopic signature of the magma^36^, while Pb isotope ratios remain unaffected by magmatic-hydrothermal processes that lead to sulfide formation^37,38^. Therefore, comparing the Pb isotope ratios of magmatic rocks with those of the ore minerals unveils metal source changes during magmatic differentiation and their consequences on SMS formation. At Kolumbo, water saturation and degassing during magmatic differentiation occurs at trachytic composition^8^. Hence, magmatic fluids should have Pb isotope ratios similar to trachyte. Injection of mafic melt into the upper magma chamber may also trigger degassing and release of magmatic fluid with basaltic-andesitic-like Pb isotope ratios (Fig. 4)^31^. In the chimney, most galena and Sb-Pb sulfosalts have trachyte-like Pb isotope ratios, indicating contribution of magmatic fluids. On the other hand, pyrite shows rhyolite-like Pb isotope ratios, but as rhyolite is degassed at Kolumbo^8,31^, only hydrothermal leaching of rhyolite may account for these Pb isotope ratios (Figs. 3B and 4). Unlike pyrite, Pb isotope ratios in galena and Sb-Pb sulfosalts spread along a mixing line between trachyte-like and rhyolite-like composition (Fig. 3B). As mafic to intermediate rocks are minor components of Kolumbo volcanic rocks, they are unlikely to control the Pb isotope ratios of the hydrothermal fluid through leaching. This diversity in Pb isotope ratios likely reflects pulses of magmatic fluids mixing with hydrothermal fluids (Fig. 3B).

### Constrains on metals sources for hydrothermal leaching

The submarine arc setting at Kolumbo, where faults crosscut the different stratigraphic units of the basin, favors hydrothermal fluid circulation through multiple potential metal sources (Fig. 1A). Modelling shows that the reaction zone – where hydrothermal leaching is most efficient^2^ – is focused to up to ~ 500 m above an intrusion, only spreading ~ 100 m laterally^39^. Hence, at Kolumbo, the reaction zone should be located between ~ 1.5 and 2.0 km depth below the crater. The lithologies at this depth are not well constrained but may correspond to the crystallized margin of the upper magma chamber within the Pelagonian Unit (Fig. 4). The rhyolite-like Pb isotope ratios of pyrite suggest that the country rocks do not provide metals to the hydrothermal fluids and support a reaction zone where rhyolitic rocks are the dominant lithology (Fig. 3B). In addition to Pb, other base metals such as Cu and Zn may be leached from rhyolite, contributing to the metal budget of the SMS^40^.

### Episodic input of magmatic fluids in the hydrothermal system

The chimneys at Kolumbo show mineralogical zoning, reflecting changes in the nature of the mineralizing fluid (Fig. 2A-D) and allowing to track changes in fluid source during the SMS formation when combined with the Pb isotope ratios of the sulfides. Pyrite is the main sulfide occurring in the chimney, especially in the ISC (Fig. 2B). According to its rhyolite-like Pb isotope ratios, hydrothermal fluids were dominant during the chimney growth (Fig. 3B). On the other hand, the Pb isotope ratios of galena and Sb-Pb sulfosalts indicate a magmatic origin (Fig. 3B). Hence, galena growth zones occurring into pyrite at the ISC-IVW transition, and galena, Sb-Pb sulfosalts occurring at the TZ and IVW (Fig. 2B and C) likely reflect pulses of magmatic fluids into the hydrothermal system. Upon magmatic degassing, As, Ag, Au, Cu, Hg, Sb, Sn and Zn preferentially partition, along with Pb, into the volatile phase at Kolumbo, either from the silicate melt or within sulfide-volatile compounds (Fig. 4)^8,15^. Systematic enrichment in Pb, Sb, Ag and Cu associated with galena layers and dense growth zones – likely nanoscale galena or Sb-Pb sulfosalts – in colloform pyrite supports repeated contribution of magmatic fluids during the chimney growth (Fig. 2D). Episodic pulses of magmatic fluids in the hydrothermal system likely contribute to the high content of epithermal elements in the Au-rich SMS at Kolumbo. The origin of the high Tl content in the SMS is uncertain. At Kolumbo, Tl is not depleted after degassing, unlike other chalcophile elements^8^, suggesting that Tl is leached from rhyolite (Fig. 4).

## Conclusion

The particular geological setting at Kolumbo volcano, with geochemically distinct country rocks and igneous rocks, allow to discriminate the sources of metals for the SMS and distinguish if they are mobilized by magmatic degassing or hydrothermal leaching using Pb isotope ratios. Here, the country rocks do not appear to contribute to the metal budget of the SMS. A single source – the igneous rocks – provides metals via two mechanisms: magmatic degassing brings epithermal and base metals, while hydrothermal leaching mobilizes Tl and likely some base metals. Additionally, specific mineral assemblages in the chimneys are associated with magmatic degassing and hydrothermal leaching, allowing us to track magmatic fluid pulses during the chimney growth. This study of Kolumbo highlights processes that cannot be easily discriminated in geochemically more homogeneous settings, such as oceanic arcs, and provides strong evidences that episodic pulses of magmatic fluids are responsible for enrichment in epithermal element during arc-SMS formation. Hydrothermal fluids leaching the subseafloor are also contributing to the metal budget of SMS and may be a major source of base metals, especially in areas with well-developed hydrothermal system and large reaction zones within country rocks.

## Electronic supplementary material

Below is the link to the electronic supplementary material.

Supplementary Material 1

Supplementary Material 2

## Data Availability

All the supporting data and methods for this study are published in the electronic supplementary material (ESM1 and ESM2, respectively).

## References

[1] Tunnicliffe, V. Hydrothermal-Vent communities of the deep sea. *Am. Sci.* **80**, 336–349 (1992).

[2] Hannington, M., de Ronde, C. E. J. & Petersen, S. Sea-Floor Tectonics and Submarine Hydrothermal Systems. in *One Hundredth Anniversary Volume* (eds. Hedenquist, J., Thompson, J. F. H., Goldfarb, R. J. & Richards, J. P.)Society of Economic Geologists, (2005). 10.5382/AV100.06

[3] Patten, C. G. C., Pitcairn, I. K., Teagle, D. A. H. & Harris, M. Mobility of Au and related elements during the hydrothermal alteration of the oceanic crust: implications for the sources of metals in VMS deposits. *Min. Deposita*. **51**, 179–200 (2016).

[4] Hannington, M. Volcanogenic massive sulfide deposits. in Treatise on Geochemistry 463–488 (Elsevier, doi:10.1016/B978-0-08-095975-7.01120-7. (2014).

[5] Fuchs, S., Hannington, M. D. & Petersen, S. Divining gold in seafloor polymetallic massive sulfide systems. *Min. Deposita*. **54**, 789–820 (2019).

[6] Li, Z. et al. Magmatic sulfide formation and oxidative dissolution in the SW Okinawa trough: A precursor to metal-bearing magmatic fluid. *Geochim. Cosmochim. Acta*. **258**, 138–155 (2019).

[7] Brandl, P. A. et al. The origin of magmas and metals at the submarine brothers volcano, kermadec Arc, new Zealand. *Econ. Geol.*10.5382/econgeo.4973 (2022).

[8] Hector, S. et al. Magmatic evolution in the Kolumbo volcano and its implication to seafloor massive sulphide formation. *Miner. Deposita* (2024).

[9] Falkenberg, J. J. et al. Pyrite trace element proxies for magmatic volatile influx in submarine subduction-related hydrothermal systems. *Geochim. Cosmochim. Acta*. **373**, 52–67 (2024).

[10] Diehl, A., de Ronde, C. E. J. & Bach, W. Subcritical Phase Separation and Occurrence of Deep-Seated Brines at the NW Caldera Vent Field, Brothers Volcano: Evidence from Fluid Inclusions in Hydrothermal Precipitates. *Geofluids* 1–22 (2020). (2020).

[11] Alt, J. C., Teagle, D. A. H., Brewer, T., Shanks, W. C. & Halliday, A. Alteration and mineralization of an oceanic forearc and the ophiolite-ocean crust analogy. *J. Geophys. Res.* **103**, 12365–12380 (1998).

[12] Patten, C. G. C. et al. Metal fluxes during magmatic degassing in the oceanic crust: sulfide mineralisation at ODP site 786B, Izu-Bonin forearc. *Min. Deposita*. **55**, 469–489 (2020).

[13] Martin, A. J. et al. Constraining Temporal variations in metal and sulfur sources using high-resolution mineral-scale analysis of pyrite: evidence from the brothers volcano, kermadec Arc, new Zealand. *Min. Deposita*. **58**, 1237–1262 (2023).

[14] Zegkinoglou, N. N. et al. Boiling-induced extreme Cu isotope fractionation in sulfide minerals forming by active hydrothermal diffusers at the Aegean Kolumbo volcano: evidence from in situ isotope analysis. *Geology* **51**, 1072–1076 (2023).

[15] Patten, C. G. C. et al. Transfer of sulfur and chalcophile metals via sulfide-volatile compound drops in the Christiana-Santorini-Kolumbo volcanic field. *Nat. Commun.* **15**, 4968 (2024). 38862488 10.1038/s41467-024-48656-9 PMC11167051

[16] Stucker, V. K., Walker, S. L., De Ronde, C. E. J., Caratori Tontini, F. & Tsuchida, S. Hydrothermal venting at hinepuia submarine volcano, kermadec Arc: Understanding Magmatic-Hydrothermal fluid chemistry. *Geochem. Geophys. Geosyst.* **18**, 3646–3661 (2017).

[17] Nomikou, P. et al. Submarine volcanoes of the Kolumbo volcanic zone NE of Santorini caldera, Greece. *Glob. Planet Change*. **90–91**, 135–151 (2012).

[18] Nomikou, P., Hübscher, C., Ruhnau, M. & Bejelou, K. Tectono-stratigraphic evolution through successive extensional events of the Anydros basin, hosting Kolumbo volcanic field at the Aegean sea. *Greece Tectonophysics*. **671**, 202–217 (2016).

[19] Heath, B. A. et al. Tectonism and its relation to magmatism around Santorini volcano from upper crustal *P* wave velocity. *JGR Solid Earth*. **124**, 10610–10629 (2019).

[20] Preine, J. et al. Spatio-temporal evolution of the Christiana-Santorini-Kolumbo volcanic field, Aegean sea. *Geology* **50**, 96–100 (2022).

[21] Sigurdsson, H. et al. Marine investigations of Greece’s Santorini volcanic field. *Eos Trans. Am. Geophys. Union*. **87**, 337–342 (2006).

[22] Kilias, S. P. et al. New insights into hydrothermal vent processes in the unique shallow-submarine arc-volcano, Kolumbo (Santorini), Greece. *Sci. Rep.* **3**, 2421 (2013). 23939372 10.1038/srep02421 PMC3741630

[23] Nomikou, P. et al. SANTORY: SANTORini’s seafloor volcanic observatory. *Front. Mar. Sci.* **9**, (2022).

[24] Li, W., Jackson, S. E., Pearson, N. J., Alard, O. & Chappell, B. W. The Cu isotopic signature of granites from the Lachlan fold belt, SE Australia. *Chem. Geol.* **258**, 38–49 (2009).

[25] Forster, M. A. & Lister, G. S. Detachment faults in the Aegean core complex of Ios, cyclades, Greece. *Geol. Soc. Lond. Special Publications*. **154**, 305–323 (1999).

[26] Flansburg, M. E., Stockli, D. F., Poulaki, E. M. & Soukis, K. Tectono-magmatic and stratigraphic evolution of the cycladic basement, Ios Island, Greece. *Tectonics* **38**, 2291–2316 (2019).

[27] Schneider, D. A., Grasemann, B., Lion, A., Soukis, K. I. & Draganits, E. Geodynamic significance of the Santorini detachment system (Cyclades, Greece). *Terra Nova*. **30**, 414–422 (2018).

[28] Soukis, K. I. & Papanikolaou, D. J. Contrasting geometry between alpine and late- to post-alpine tectonic structures in Anafi Island (Cyclades). *Geosociety* **36**, 1688 (2004).

[29] Hübscher, C., Ruhnau, M. & Nomikou, P. Volcano-tectonic evolution of the polygenetic Kolumbo submarine Volcano/Santorini (Aegean Sea). *J. Volcanol. Geoth. Res.* **291**, 101–111 (2015).

[30] Cantner, K., Carey, S. & Nomikou, P. Integrated volcanologic and petrologic analysis of the 1650AD eruption of Kolumbo submarine volcano, Greece. *J. Volcanol. Geoth. Res.* **269**, 28–43 (2014).

[31] Klaver, M. et al. A distinct source and differentiation history for Kolumbo submarine volcano, Santorini volcanic field, Aegean Arc. *Geochem. Geophys. Geosystems: G(3)*. **17**, 3254–3273 (2016). 10.1002/2016GC006398 PMC5114867 27917071

[32] Chrapkiewicz, K. et al. Magma chamber detected beneath an Arc volcano with Full-Waveform inversion of Active‐Source seismic data. *Geochem. Geophys. Geosystems: G*. **3**, 23 (2022).

[33] Nomikou, P., Hübscher, C. & Carey, S. The Christiana–Santorini–Kolumbo volcanic field. *Elements* **15**, 171–176 (2019).

[34] Klaver, M. *Dynamics of Magma Generation and Differentiation in the central-eastern Aegean Arc* (Vrije Universiteit Amsterdam, 2016).

[35] White, W. M. *Isotope Geochemistry* (Wiley Blackwell, 2015).

[36] Hofmann, A. W. Mantle geochemistry: the message from oceanic volcanism. *Nature* **385**, 219–229 (1997).

[37] Zeng, Z. et al. Sulfur and lead isotopic compositions of massive sulfides from deep-sea hydrothermal systems: implications for ore genesis and fluid circulation. *Ore Geol. Rev.* **87**, 155–171 (2017).

[38] Zametzer, A. et al. Applications of Pb isotopes in granite K-feldspar and Pb evolution in the Yilgarn craton. *Geochim. Cosmochim. Acta*. **320**, 279–303 (2022).

[39] Scott, S., Driesner, T. & Weis, P. Geologic controls on supercritical geothermal resources above magmatic intrusions. *Nat. Commun.* **6**, 7837 (2015). 26211617 10.1038/ncomms8837 PMC4525172

[40] Barriga, F. J. A. S. & Fyfe, W. S. Multi-phase water-rhyolite interaction and ore fluid generation at Aljustrel, Portugal. *Miner. Deposita*. **33**, 188–207 (1997).

