# Supplementary material for: Unravel subseafloor hydrothermal leaching and magmatic degassing during chimney formation at Kolumbo volcano
Source: Sci Rep. 2025 Apr 26;15:14673. doi: 10.1038/s41598-025-99586-5 (PMC12033240; doi:10.1038/s41598-025-99586-5)
Supplement: Supplementary file 2 — Supplementary Material 2 [file 41598_2025_99586_MOESM2_ESM.docx]

# Supplementary material 2 (ESM2)

## Methods

### Whole rock geochemistry

Chemical analyses were performed at the Laboratory for Environmental and Raw Materials Analysis at the Institute of Applied Geosciences in Karlsruhe, Germany, with the exception of Hg content measurement by atomic absorption spectroscopy at the Oceanic and Continental Environment and Paleoenvironment mixed research unit (UMR EPOC) in Bordeaux, France. Major element compositions were measured by X-ray fluorescence (XRF; S4 Explorer, Bruker AXS) on glass beads with BHVO-1, MRG-1, RGM-1 and SY-2 as reference materials, as a measure of analytical precision and accuracy (see ESM1). Carbon and sulfur content were measured on rock powder by solid state infrared absorption using a Carbon-Sulphur Analyzer (CS-2000, Eltra) with steel (92400-3050), ductile iron (92400-3100) and barium sulphate (90821) standards from Eltra as reference material as a measure of analytical precision and accuracy (see ESM1). Trace element contents were measured by laser-ablation inductively-coupled-plasma mass-spectrometry (LA-ICP-MS) on pressed powder pellets using a Teledyne 193 nm Excimer Laser coupled to an ICP-MS (Element XR ThermoFisher) with spot size of 85 µm, laser frequency of 10 Hz, fluence of 5 J.cm^-2^, He, Ar and N flow of 0.3 L.min^-1^, 0.85 L.min^-1^ and 10 mL.min^-1^, respectively. Pressed powder pellets are prepared following the method described in Patten et al. (2023). Calibration and data quality checking was done using pressed powder pellets of standards BHVO-1, BHVO-2, BCR-2 and BIR-1 from the USGS. In order to improve data quality, Au contents were measured a second time, on the same equipment, following the method of ultra-low detection of Au on pressed powder pellets by LA-ICP-MS developed by Patten et al. (2023). Calibration and data quality checking was done using pressed power pellets of standards BHVO-2, BCR-2, BIR-1 from the USGS, MRG-1 and TDB-1 from NRCan and TSD-41, an in-house epidosite standard. Data reduction of all LA-ICP-MS analysis was done using the Iolite software 3DRS plugin v.4.8.3 (Paton et al., 2011). Accuracy and precision for reference materials (<15% for most elements) as well as limits of detections are detailed in Supplementary data (see ESM1). The Hg content in the samples was measured by spectrophotometry with a direct Hg analyser DMA-80 (Milestone) using standards BCR-277R and TCEGir (in-house) for calibration (see ESM1).

### Pb isotope determination

Radiogenic Pb isotope ratios (^206^Pb/^204^Pb, ^207^Pb/^204^Pb, ^208^Pb/^204^Pb) of 39 bulk rocks (see ESM1) were measured at the Department of Earth Sciences, University of Geneva, Switzerland, following the method described in detail in Chiaradia et al. (2020). About 150 mg of whole rock powder were dissolved during 7 days in Savillex® Teflon vials using 4 ml of concentrated HF and 1 mL of HNO_3_, at a temperature of 140 °C and with the help of ultrasonication for 30 minutes twice a day. Subsequently, samples were dried and re-dissolved for 3 days (also with 30 minutes ultrasonication twice a day) in 3 ml of HNO_3_ and dried again. Lead was separated from the matrix using micro-columns loaded with AG-MP1-M resin in a hydrobromic medium. The purified Pb was redissolved in a 2% HNO_3_ solution spiked with a Tl standard. Isotope ratios were measured using a Thermo Neptune PLUS Multi-Collector ICP-MS in static mode. Instrumental fractionation was corrected in-run by using ^203^Tl/^205^Tl = 0.418922 and the ^204^Hg interference on ^204^Pb was corrected by monitoring ^202^Hg. The SRM981 standard was used to check the long-term external reproducibility of measures which is 0.0079% for ^206^Pb/^204^Pb, 0.0070% for ^207^Pb/^204^Pb, 0.0093% for ^208^Pb/^204^Pb, 0.0059 for ^208^Pb/^206^Pb, 0.0057% for ^207^Pb/^206^Pb, and 0.0041% for ^208^Pb/^207^Pb. The in-run corrected Pb isotope ratios were further corrected for external fractionation (due to a systematic difference between measured and accepted ratios of SRM981) by a value of +0.36‰ amu using the SRM981 values proposed by Baker et al. (2004). Procedural blanks are <200 pg and are insignificant compared to the amount of sample Pb.

### In-situ determination of Pb isotope

Radiogenic isotope ratios of Pb (^206^Pb/^204^Pb, ^207^Pb/^204^Pb, ^208^Pb/^204^Pb, ^207^Pb/^206^Pb, ^208^Pb/^206^Pb) of ore minerals (pyrite, galena, Sb-Pb sulfosalts; see ESM1) were measured at the Geological Survey of Finland (Espoo, Finland) using a Nu Plasma HR multicollector ICP-MS (Nu Instruments Ltd., UK) in combination with an Analyte G2 laser ablation system (Photon Machines, USA). Galena, pyrite and Sb-Pb sulfosalts were investigated by in-situ LA-ICP-MS analysis with spot size of 30 µm for pyrite and 6 x 100 μm lines for galena and Sb-Pb sulfosalts, laser frequency of 3 Hz, fluence of 3 J·cm^-2^, He and Ar flow of 0.4 L·min^-1^ and 0.1 L·min^-1^, respectively. The mass discrimination factor for Pb was determined using a Tl solution nebulized at the same time as the sample using a desolvating nebulizer. Broken Hill galena was used to monitor the precision and accuracy of the measurements. The obtained average accuracy is below 0.34‰ for ^207^Pb/^206^Pb and 0.08‰ for ^208^Pb/^206^Pb, compared with the certified value of Townsend et al. (1998). Due to high amounts of Hg in the analyzed pyrites, the isobaric interference of ^204^Hg on ^204^Pb could not be sufficiently corrected and therefore only ^207^Pb/^206^Pb and ^208^Pb/^206^Pb values are reported for pyrites. The Pb–Pb isotope measurement included a baseline measurement for 1 minute prior to the sample batch measurements. Each isotope ratio measurement consists of 45 seconds of data acquisition during the laser ablation.

### Micro-XRF

Micro-XRF mapping of the Kolumbo chimney sample was performed at Innsbruck University using the Bruker M4 Tornado. A voltage of 50 kV and a current of 600 µA was used to provide optimal excitation and detectability of the elements. The sample was analyzed under vacuum conditions to enhance the detection of light elements and minimize signal attenuation. An area of approximately 1998x1206 pixels was scanned with a step size of 50 µm and an acquisition time of 20 ms/pixel. The fluorescence signal was captured by two silicon drift detectors, and data processing was carried out in the Bruker M4 software.

### EDX elemental linescan

The EDX elemental linescan was performed at the Laboratory for Environmental and Raw Materials Analysis in Karlsruhe, using an Oxford AZtecOne EDX detector coupled to a scanning electron microscope Hitachi FlexSEM1000II. A voltage of 20kV and a spot intensity of 80 was used to provide optimal excitation and detectability of the elements. The sample was analyzed under vacuum conditions to enhance the detection of light elements and minimize signal attenuation. The data processing was carried out using the AZtecLiveOne software from Oxford. Element signal smoothing was done using the adjacent averaging method (10 neighbors’ values) with the Origin software to reduce the signal noise and improve readability.

### Monte Carlo-based modeling of an assimilation–fractional crystallization process

Kolumbo volcanic rocks follows a trend of increasing ^207^Pb/^206^Pb and ^208^Pb/^206^Pb values as the magma evolves to felsic compositions that we interpret as the result of assimilation of Cycladic Basement during magmatic differentiation (Fig. 3B). In order to quantify the assimilation process, we modeled crustal assimilation using the plot ^207^Pb/^206^Pb versus Rb, because Rb is a typical highly incompatible element during magmatic fractionation and the ^207^Pb/^206^Pb ratio reflects well the input of crustal material (high ^207^Pb/^206^Pb values) versus a mantle-derived magma (low ^207^Pb/^206^Pb values) (Fig. 4). Increasing Rb contents of magmatic rocks correlates well with increasing ^207^Pb/^206^Pb values, suggesting an assimilation–fractional crystallization process that we have modelled using DePaolo’ s equations (DePaolo, 1981). Because several of the parameters used in these equations are more or less well constrained we have used a Monte Carlo approach in which we have let variables to range within geologically and geochemically constrained intervals (Table 1). Monte Carlo simulations of the model explain the greatest majority of the Kolumbo volcanic rock compositions and imply a maximum assimilation of Cycladic basement between 20 and 40%, in the most evolved, rhyolitic, volcanic rocks (Fig. 3C). The model also returns a most probable assimilation rate (r value in DePaolo’s equations) between 0.4 and 0.75, which corresponds to mid- to deep crustal levels (DePaolo, 1981), and to ^207^Pb/^206^Pb values of ~0.838 for the assimilant, consistent with the Cycladic Basement. This scenario is consistent with available data on the Kolumbo magmatic plumbing system, according to which basaltic-andesitic magma evolves to rhyolitic composition in a lower crustal magma chamber at the base of the crust before rising toward an upper magma chamber at 2-4 km depth (Klaver et al., 2016).

Table 1 : Range of values used in to model assimilation–fractional crystallization processes (DePaolo, 1981) at Kolumbo, following a Monte Carlo approach.

| **Parameter** | **Description** | **Minimum** | **Maximum** |
| --- | --- | --- | --- |
| DPb | Range of bulk rock partition coefficient for Pb | 0.1 | 0.25 |
| rv | Assimilation rate | 0.05 | 0.80 |
| Pb_par | Range of variability of Pb content in parent magma | 4 ppm | 4 ppm |
| Pb_ass | Range of variability of Pb content in assimilant | 10 ppm | 40 ppm |
| Pbiso_par | Range of variability of ^207^Pb/^206^Pb value in parent magma | 0.826 | 0.828 |
| Pbiso_ass | Range of variability of ^207^Pb/^206^Pb value in assimilant | 0.835 | 0.855 |
| DRb | Range of bulk rock partition coefficient for Rb | 0 | 0.1 |
| Rb_par | Range of variability of Rb content in parent magma | 10 ppm | 15 ppm |
| Rb_ass | Range of variability of Rb content in assimilant | 80 ppm | 120 ppm |
| zPb | zPb parameter in DePaolo's (1981) equations | (rv+DPb-1)/(rv-1) | |
| zRb | zRb parameter in DePaolo's (1981) equations | (rv+DRb-1)/(rv-1) | |

**References**

Baker, J., Peate, D., Waight, T., and Meyzen, C., 2004, Pb isotopic analysis of standards and samples using a 207Pb–204Pb double spike and thallium to correct for mass bias with a double-focusing MC-ICP-MS: Chemical Geology, v. 211, p. 275–303, doi:10.1016/j.chemgeo.2004.06.030.

Chiaradia, M., Müntener, O., and Beate, B., 2020, Effects of aseismic ridge subduction on the geochemistry of frontal arc magmas: Earth and Planetary Science Letters, v. 531, p. 115984, doi:10.1016/j.epsl.2019.115984.

DePaolo, D.J., 1981, Trace element and isotopic effects of combined wallrock assimilation and fractional crystallization: Earth and Planetary Science Letters, v. 53, p. 189–202, doi:10.1016/0012-821X(81)90153-9.

Klaver, M., Carey, S., Nomikou, P., Smet, I., Godelitsas, A., and Vroon, P., 2016, A distinct source and differentiation history for Kolumbo submarine volcano, Santorini volcanic field, Aegean arc: Geochemistry, geophysics, geosystems : G(3), v. 17, p. 3254–3273, doi:10.1002/2016GC006398.

Paton, C., Hellstrom, J., Paul, B., Woodhead, J., and Hergt, J., 2011, Iolite: Freeware for the visualisation and processing of mass spectrometric data: Journal of Analytical Atomic Spectrometry, v. 26, p. 2508, doi:10.1039/c1ja10172b.

Patten, C.G.C., Beranoaguirre, A., Hector, S., Gudelius, D., Kolb, J., and Eiche, E., 2023, Improved whole rock low detection limit gold analysis by LA-ICP-MS utilizing pressed-powder-pellets: International Journal of Mass Spectrometry, p. 117039, doi:10.1016/j.ijms.2023.117039.

Townsend, A.T., Yu, Z., Mcgoldrick, P., and Hutton, J.A., 1998, Precise lead isotope ratios in Australian galena samples by high resolution inductively coupled plasma mass spectrometry: Journal of Analytical Atomic Spectrometry, v. 13, p. 809–813, doi:10.1039/A801397G.
